# Pediatric polysomnography-flagging etiologies and impact on the clinical timeline

**DOI:** 10.3389/frsle.2023.1302509

**Published:** 2024-01-18

**Authors:** Seema Rani, John Schanz, Kapil Chauhan, August Kolb, Victoria Gatta, Abigail Strang, Aaron C. Chidekel

**Affiliations:** ^1^Department of Pulmonary Medicine, Nemours Children's Health, Wilmington, DE, United States; ^2^Department of Surgery, Johns Hopkins Hospital, Baltimore, MD, United States; ^3^Howard University College of Medicine, Washington, DC, United States

**Keywords:** polysomnography, abnormal sleep studies, expedited review, pediatrics, obstructive sleep apnea

## Abstract

**Background/objective:**

There is a paucity of literature regarding “flagging” abnormal sleep studies for expedited review. This single-center retrospective analysis (*n* = 266) of flagged polysomnography studies from 2019 to 2022 aimed to investigate flagging and its impact on the clinical timeline.

**Methods:**

Two hundred sixty-six flagged polysomnography studies from 2019 to 2022 were retrospectively reviewed.

**Results:**

Flagged study etiologies included repetitive brief oxygen desaturations (46.6%), sustained desaturations (32.3%), sustained hypercapnia (5.6%), or other concerning events (15.5%). The median time between a flagged study and scoring report finalization, medical intervention, and surgical intervention were 0 (2) days, 2 (3) days, 5 (11.25) days, and 44 (73) days, respectively. Patients with apnea–hypopnea index >30 had less time between a flagged study and surgical intervention (65.3 ± 96.7 days vs. 112 ± 119 days, *p* = 0.044).

**Conclusion:**

As anticipated, the time to surgical intervention was longer than to medical intervention. Patients with a higher disease severity experienced quicker scoring, report finalization, and surgical intervention.

## 1 Introduction

Untreated pediatric obstructive sleep apnea (OSA) syndrome has been well documented to have long-term sequelae involving multiorgan system dysfunction (Schechter, 2002; Kheirandish-Gozal and Gozal, 2017). Manifestations include autonomic and endothelial dysfunction, cardiac remodeling (Ingram et al., 2017; Baker-Smith et al., 2021), insulin resistance, dyslipidemia, metabolic derangements (Blechner and Williamson, 2016), negative associations with longitudinal growth (Park et al., 2018), impaired neurocognition, cortical thinning, and impaired attention (Thomas et al., 2022). However, these sequelae may not be permanent.

Recent research has found that early recognition and appropriate surgical treatment to improve respiration during sleep can lower the risk of consequences such as pulmonary hypertension, cor pulmonale (Baker-Smith et al., 2021), and insulin resistance (Bhatt et al., 2021). Other studies have documented improvements in neuropsychological testing in children with OSA syndrome postsurgical interventions such as tonsillectomy and adenoidectomy (Yu et al., 2017). Earlier detection may present the opportunity for intervention for overall improved health quality and health outcomes in pediatric patients, but future research is necessary.

In the presence of significant sleep-related physiologic abnormalities or other concerning events encountered during a polysomnography, accredited sleep laboratories and performing technologists utilize a standardized protocol with the involvement of an on-call supervising physician for flagging a study for expedited scoring and review. While there is literature pertaining to sleep emergencies (Thomas and Chang-Ho, 2011; Collop, 2021), no studies report specific criteria for the expedited scoring and interpretation of pediatric polysomnographies or their potential association with treatment timing.

Despite the growing evidence supporting earlier interventions for pediatric OSA management (Marcus et al., 2012, 2013), there is a dearth of research discerning a standard timeline for definitive management. Without exploring these timelines, the optimal management of sleep-related respiratory disturbances in children cannot be adequately assessed.

The present study aimed to explore pediatric OSA syndrome by providing data on the impact of polysomnography flagging and the associated timelines on clinical management.

## 2 Methods

This was a single-center retrospective chart review study conducted at Nemours Children's Hospital in Delaware, a pediatric tertiary care center, with a defined study period from 2019 through 2022. All data collection and protocols were approved by the Nemours institutional review board. All pediatric patients who had a polysomnography flagged for expedited review during the study period were included. Studies were flagged based on established laboratory protocols and at the discretion of the on-call physician. All data including basic demographic, clinical, and polysomnography parameters were obtained from the electronic medical record. The baseline characteristics included age, race, ethnicity, sex at birth, body mass index, and medical comorbidities.

The selected polysomnography parameters included total sleep time (TST), sleep efficiency, apnea–hypopnea index (AHI), OSA index, arousal index, and minimum oxygen saturation (SpO_2_), as well as the presence of hypoventilation (end-tidal carbon dioxide [EtCO_2_] > 50 torr for at least 25% of TST) and hypoxia (SpO_2_ < 90% for at least 5% of TST). The reasons for polysomnography flagging were obtained from the sleep laboratory database by an experienced sleep medicine technologist.

The dates of the polysomnography encounter, report flagging, scoring, clinical physician encounter note finalization, and any subsequent medical or surgical intervention were recorded. Medical intervention was defined as the ordering of nocturnal supplemental oxygen therapy or positive airway pressure with either continuous positive airway pressure (CPAP) or bilevel positive airway pressure. Surgical intervention included adenoidectomy, tonsillectomy, and adenotonsillectomy. Patients who had received prior airway surgery were not coded in the surgical group unless they received a new or revised surgical procedure in response to the flagged polysomnography study. Notation was made of patients admitted overnight after surgery due to new oxygen or CPAP requirements in the immediate postoperative period.

Statistical analyses were conducted using Student's *t*-test for continuous variables and both the chi-square and Fisher's exact tests for categorical variables. Further tests included the Mann–Whitney *U*-test to assess for impact of skew on non-parametric data. Statistical significance was defined as *p* < 0.05. Statistical analyses were performed using SPSS Statistics version 27 (IBM Corp., Armonk, NY) and Microsoft Excel version 16.60 (Redmond, WA).

## 3 Results

The demographic, clinical, and polysomnography data of the pediatric patients with flagged polysomnographies are illustrated in Table 1. Most patients were white non-Hispanic males. The most common pulmonary, cardiac, genetic, and neuromuscular comorbidities were asthma (20.7%), congenital heart disease (22.9%), syndromic chromosomal abnormalities (14.3%), and cerebral palsy (7.9%), respectively. Notably, 30.1% of patients had a history of airway surgery prior to their flagged polysomnography, with 70% of those surgeries being adenotonsillectomy. Patients older than 8 years had higher rates of prior airway surgery compared with those younger than 8 years (43.7% vs. 21.5%, *p* < 0.0001).

**Table 1:** Baseline characteristics and polysomnography data.

**Characteristic**	**Value**
**Demographics**	
Race, *n* (%)	
Black/African American	102 (38.3)
White/Caucasian	131 (49.2)
Other	33 (12.4)
Ethnicity, *n* (%)	
Non-Hispanic/Latino	232 (87.2)
Another Hispanic/Latino/Spanish	17 (6.4)
Other	17 (6.4)
Male, *n* (%)	178 (66.9)
Age, years	6.92 ± 6.02
Body mass index, kg/m^2^	23.3 ± 11.6
Comorbid conditions, *n* (%)	
Pulmonary	110 (41.4)
Cardiac	62 (23.3)
Genetic	81 (30.5)
Neuromuscular	31 (11.7)
Obesity	62 (23.3)
History of prior airway surgery	80 (30.1)
Polysomnography flagging reasons, *n* (%)	
Sustained desaturations < 85%	86 (32.3)
Repetitive brief desaturations < 80%	124 (46.6)
Sustained end-tidal carbon dioxide > 60 torr	15 (5.6)
Other concerning events	41 (15.5)
Polysomnography parameters	
Total sleep time, minutes	372 ± 86.9
Sleep efficiency, %	79.3 ± 14.3
Apnea index, events/hour	14.7 ± 17.7
Obstructive apnea index, events/hour	9.8 ± 14.9
Apnea hypopnea index, events/hour	47.2 ± 36.1
Oxygen saturation nadir, %	76.5 ± 9.87
Time < 90% oxygen saturation, %	8.6 ± 15.5
Peak end-tidal carbon dioxide, torr	57.7 ± 7.46
Time end-tidal carbon dioxide > 50 torr, %	25.1 ± 30.6
Arousal index, number of hours sleep	40.7 ± 31.6
Treatment methods, *n* (%)	
Medical	108 (40.6)
Surgical	101 (37.9)
Adenoidectomy	10 (9.9)
Tonsillectomy	8 (7.9)
Adenotonsillectomy	83 (82.2)

Values are reported as *n* (%) or mean ± *SD* unless otherwise specified.

The mean TST, sleep efficiency, and AHI were 372 ± 86.9 minutes, 79.3 ± 14.3%, and 47.2 ± 36.1 events per hour, respectively. The mean percentage of TST with SpO_2_ < 90% and EtCO_2_ > 50 torr were 8.60 ± 15.5% and 25.1 ± 30.6%, respectively.

Polysomnographies were flagged most frequently secondary to repetitive, brief desaturations with SpO_2_ < 80% (46.6%), sustained desaturations with SpO_2_ < 85% (32.3%), and other concerning events (15.5%), as well as sustained EtCO_2_ > 60 torr (5.6%). The prevalence of sleep studies that met diagnostic criteria for nocturnal hypoxemia (35.0%) and hypoventilation (36.8%) were similar.

**Figure 1:**
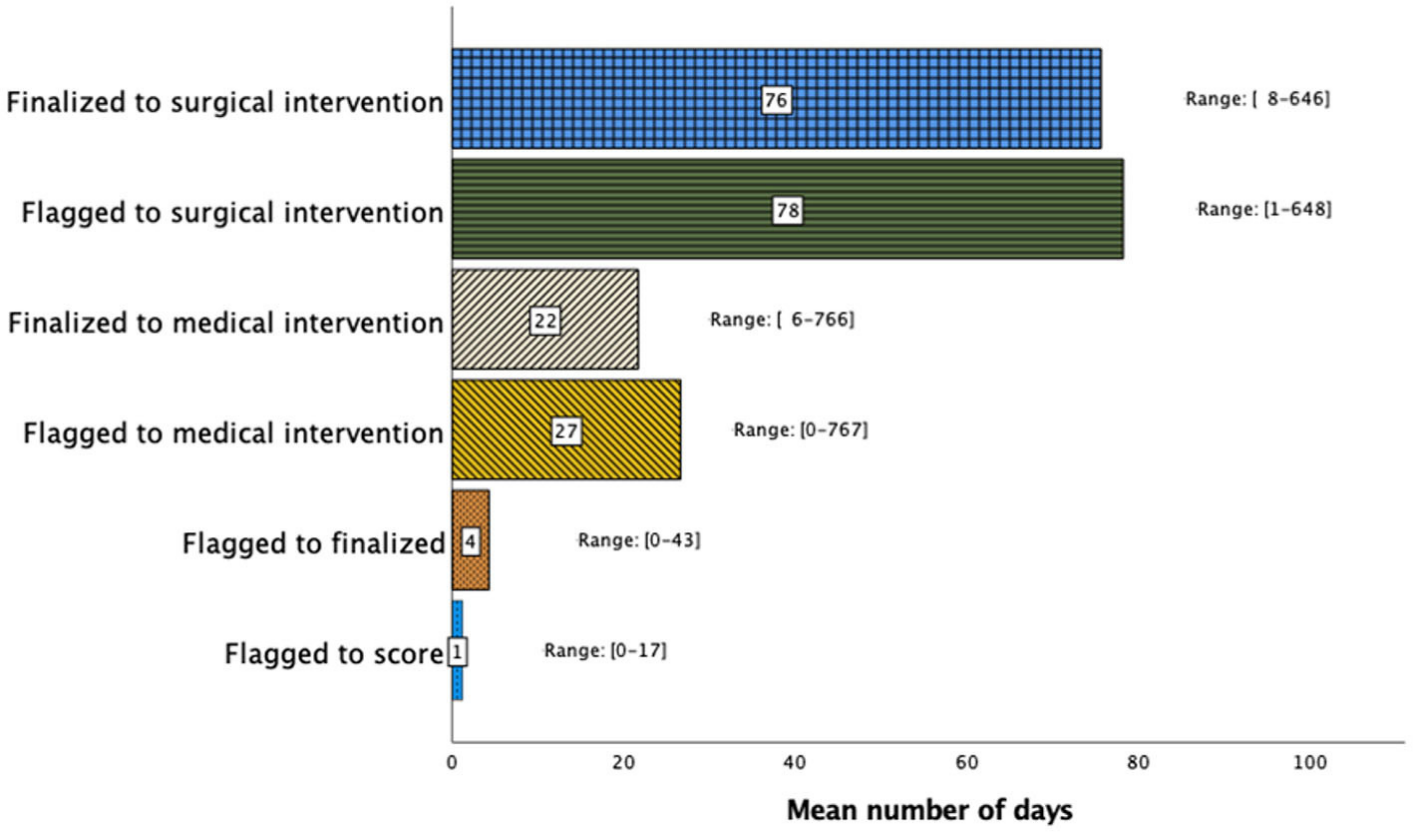
Sleep study clinical timeline demonstrating the timing interval (days) between study flagging, scoring, report finalization, medical intervention, and surgical intervention. Although the time between flagging and report finalization was short, the time to medical or surgical treatment varied widely.

The mean times between a flagged study and scoring, report finalization, medical intervention, and surgical intervention were 1.18 ± 2.09 days, 4.29 ± 6.01 days, 26.6 ± 93.0 days, and 78.2 ± 105 days, respectively, as shown in Figure 1. Subgroup analysis revealed that patients with a greater disease severity characterized by AHI > 30 experienced shorter mean intervals between study flagging and scoring (0.970 vs. 1.49 days, *p* = 0.048), report finalization (3.42 vs. 5.61 days, *p* = 0.005), and surgical intervention (65.3 vs. 112 days, *p* = 0.044).

**Figure 2:**
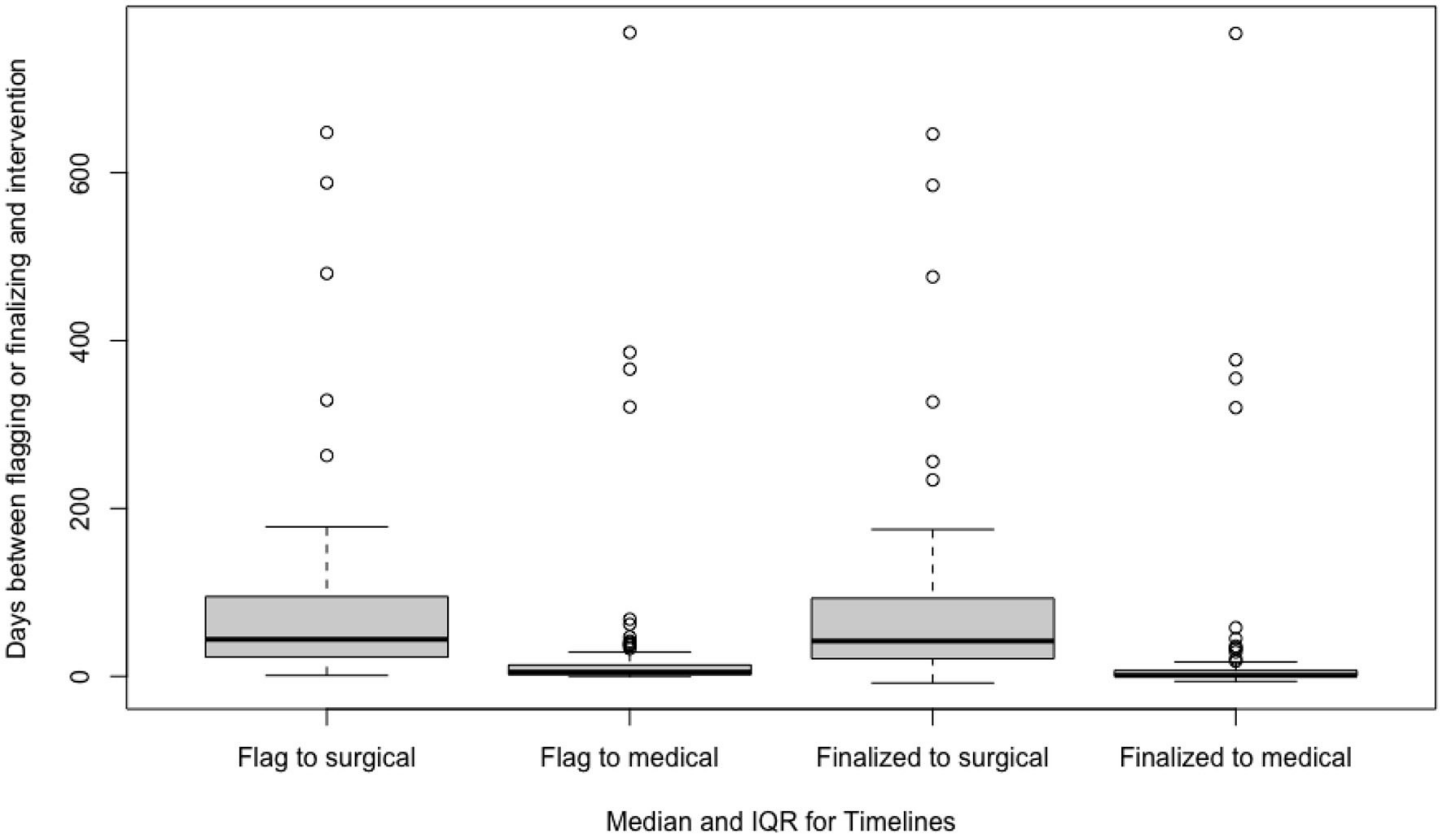
Median time and interquartile ranges between a flagged study to scoring, report finalization, medical intervention, and surgical intervention.

The median time between a flagged study and scoring, report finalization, medical intervention, and surgical intervention were 0 (2) days, 2 (3) days, 5 (11.25) days, and 44 (73) days, respectively. The median time and interquartile ranges for timelines are depicted in Table 2 and Figure 2. Subgroup analysis using the Mann–Whitney *U*-test of patients with greater disease severity characterized by AHI > 30 experienced shorter median intervals between study flagging and scoring (*p* = 0.033) and surgical intervention (*p* = 0.001). This analysis was not statistically significant for the time between study flagging and report finalization (*p* = 0.215) and medical intervention (*p* = 0.992).

**Table 2:** Descriptive statistics of sleep report flagging, finalization, and intervention.

**Characteristic**	**Value**
Time between study flagging and scoring	0 (2)
Time between study flagging and finalization	2 (3)
Time between study flagging and medical intervention	5 (11.25)
Time between study finalization and medical intervention	1 (7)
Time between study flagging and surgical intervention	44 (71.5)
Time between study finalization and surgical intervention	42 (72)

Values are reported as median (interquartile range) unless otherwise specified.

There were similar proportions of surgical (37.9%) and medical (40.6%) treatment interventions following flagged polysomnographies. Of those who underwent surgery, with adenotonsillectomy (82.1%) being the most common, 36.6% required an overnight admission due to a new CPAP or oxygen requirement.

## 4 Discussion

The most frequent reason for flagging polysomnographies was repetitive brief oxygen desaturations. The frequency of surgical interventions and medical treatment interventions following a flagged study were similar, although, on average, medical treatment occurred more quickly than surgical intervention. Adenotonsillectomy was the most frequently employed surgical treatment method compared with adenoidectomy and tonsillectomy alone.

Patients with higher disease severity experienced quicker scoring, report finalization, and surgical intervention, which may prevent or attenuate consequent sequelae. It is important to note that there may have been skew in these averages as the analyses of the median did not provide statistical significance for report finalization and medical intervention. This study is novel in its contribution of a 3-year management timeline after flagging a polysomnography, thereby creating a foundation for assessing future interventions to expedite treating pediatric OSA.

There are several limitations to this study. Namely, this study examines only a single center's approach, and there was no control group depicting the timelines of the studies that were not flagged. The authors did not further explore the ramifications between earlier interventions vs. later interventions regarding flagged polysomnographies. These weaknesses should inform future research on the management of polysomnographies. Future directions should include the establishment of protocols to facilitate streamlined care between the sleep laboratory and both the surgical and medical teams. Investigations into the efficacy of these established algorithms also warrant future examination. Pandemic staffing shortages, with operating theaters being shut for days, and recent positive airway pressure machine recalls may have impacted the timeline in both interventions. Therefore, more studies that include a comparison group for timeline differences in the post-pandemic period may provide additional insights.

In conclusion, the most frequent reason for flagging polysomnographies was repetitive brief oxygen desaturations. The mean AHI of the cohort was in the severe OSA range. Adenotonsillectomy was the most frequently performed surgery. As hypothesized in our review of flagged polysomnography studies, the time to surgical intervention was longer than medical intervention. Patients with higher disease severity experienced quicker scoring, report finalization, and surgical interventions.

## Data availability statement

The raw data supporting the conclusions of this article will be made available by the authors upon request.

## Ethics statement

The studies involving humans were approved by Nemours Institutional Review Board, Nemours Children's Health. The studies were conducted in accordance with the local legislation and institutional requirements. The ethics committee/institutional review board waived the requirement of written informed consent for participation from the participants or the participants' legal guardians/next of kin because this study was a retrospective chart review and all data has been de-identified.

## Author contributions

SR: Conceptualization, Data curation, Investigation, Project administration, Supervision, Validation, Visualization, Writing—original draft, Writing—review & editing. JS: Data curation, Formal analysis, Methodology, Software, Writing—original draft, Writing—review & editing. KC: Software, Writing—original draft, Writing—review & editing. AK: Data curation, Formal analysis, Methodology, Resources, Software, Writing—review & editing. VG: Resources, Software, Writing—review & editing. AS: Conceptualization, Investigation, Methodology, Supervision, Validation, Writing—review & editing. AC: Conceptualization, Resources, Supervision, Validation, Visualization, Writing—review & editing.
